# HIF-1*α* Regulated WTAP Overexpression Promoting the Warburg Effect of Ovarian Cancer by m6A-Dependent Manner

**DOI:** 10.1155/2022/6130806

**Published:** 2022-06-12

**Authors:** Yuanyuan Lyu, Yilin Zhang, Yuhan Wang, Yonghong Luo, Huafeng Ding, Peiling Li, Guantai Ni

**Affiliations:** ^1^Department of Obstetrics and Gynecology, The First Affiliated Hospital of Wannan Medical College, Wuhu, Anhui 241001, China; ^2^Department of Obstetrics and Gynecology, The Second Affiliated Hospital of Harbin Medical University, Harbin, 150086 Heilongjiang, China

## Abstract

N6-methyladenosine (m6A) RNA methylation has been determined to execute crucial functions in tumorigenesis and cancer development. WT1-associated protein (WTAP) has an important “writer” role in m6A modification, and it is also a nuclear protein that colocalizes with splicing factors and plays a critical role in cell function and cancer progression. However, little is known about the role of WTAP in ovarian cancer (OC) and its mechanisms. In this study, we found for the first time that hypoxia-inducible factor (HIF)-1*α* could positively regulate increased expression of WTAP under hypoxia. And further results revealed that WTAP expression was closely associated with the clinicopathological features of OC, and high expression of WTAP predicted low survival rate in patients with OC. In addition, cell proliferation and invasive capacity were significantly reduced after knockdown of WTAP expression in OC cells. However, cell proliferation and invasive ability were significantly enhanced after overexpression of WTAP. Additionally, we find that WTAP interacts with DGCR8 (a crucial chip protein) to regulate the expression of microRNA-200 (miR-200) in an m6A-dependent way. Further experiments showed that the key glycolysis enzyme HK2 could be positively regulated by miR-200, which significantly affected the intracellular Warburg effect. In conclusion, this is considered uncovered that upregulation of WTAP expression by HIF-1*α* intercedes with miRNA processing, accelerates the Warburg impact, and advances the event and advancement of tumor, thus giving a novel viewpoint on m6A adjustment in OC movement.

## 1. Introduction

Ovarian cancer (OC) is the foremost deadly gynecologic danger influencing ladies, and it right now positions fifth in cancer-related passing among ladies worldwide [1, 2]. Even though advance has been made in treating early-stage OC, there is no compelling procedure to avoid or treat dangerous expansion and metastasis, the major causes of OC-related mortality [3, 4].

Hypoxia is an essential component in the tumor microenvironment and is one of the most common conditions driving tumor formation [5, 6]. Hypoxia can make tumors present a more extreme phenotype and increase the invasiveness and diffusion of tumors that enhance proliferation and metastasis, thus reducing cancer patients' survival rate [7, 8]. Numerous studies have reported that the expression of many genes in the microenvironment of tumor growth is subject to hypoxia-inducible factor- (HIF)-1*α*-activated transcription. HIF-1*α* has a very important role in tumor growth, angiogenesis, proliferation, motility, invasion, and metastasis [9, 10]. Intratumoral hypoxia is also reported as a common finding in advanced OC [11]. It has shown that hypoxia affects OC cell invasion and adhesion function by regulating ErbB signaling abnormality, thus promoting cell proliferation. This may be regulated by the HIF-1*α*-TGFA-EGFR-ErbB2-MYC axis [12]. Another study showed that hypoxia regulates DPP4 expression, protein hydrolytic inactivation, and shedding in OC cells [13].

In currently known eukaryotic organisms, studies have found that the regulation of messenger RNAs (mRNAs) by N6-methyladenosine (m6A) modifications is very common and generally effective [14–16]. An increasing number of studies have found that a variety of cellular biological functions are mostly regulated by the expression level of m6A [17]. For example, m6A can significantly affect the transcriptional splicing, nuclear RNA output, and protein translation control processes of cells [18, 19]. The identification of WT1-associated protein (WTAP), methyltransferase-like (METTL)14, and METTL3 as the three main methylases of m6A modification has been repeatedly confirmed and reported [20, 21]. Aberrant regulation of m6A methylation modification has been associated with the development of many human cancers. In particular, regulation of the microRNA (miRNAs) maturation process can lead to tumorigenesis when abnormal regulation occurs [22]. For example, in hepatocellular carcinoma, METTL14 can regulate the maturation of many essential miRNAs in cells through m6A modifications, thereby inhibiting the metastatic ability of hepatocellular carcinoma cells [23]. Most recent investigation uncovers that METTL3 promotes tumor expansion of bladder cancer by quickening price-miR-221/222 development in an m6A-dependent way [24]. Zhang et al. also reported in the literature that miR-425-5p significantly inhibits WTAP expression in acute leukemia. And overexpression of WTAP reversed the inhibitory effects of miR-425-5p on AML cell proliferation, apoptosis, and migration and invasion [25]. Numerous studies have confirmed that the miR-200 family is highly expressed in OC and can be used as a biomarker for the diagnosis and prognosis of OC [26]. However, there is no report on whether WTAP can regulate miR-200, which raises our concern.

The Warburg effect is that cancer cells grow rapidly, and this rapid growth puts the cells in a constant state of hypoxia, so the cancer cells shut down the aerobic oxidation that requires mitochondria, and energy is obtained through anaerobic glucose enzymes [27]. The Warburg effect is more common in tumors and is mainly involved in promoting cell proliferation and invasion and migration in tumor cells [24, 28]. HK2 plays an essential role in glycolysis [24]. Based on the studies, we propose a novel important regulatory axis of HIF-1*α*-WTAP-miR-200-HK2 which accelerates the Warburg effect in OC.

## 2. Materials and Methods

### 2.1. Cell Culture

We purchased human OC cell lines A2780, 3AO, SKOV3 OVCAR3, HO8910, and OVCAR8 from the National Collection of Authenticated Cell Culture (Shanghai, China) and cultured the different cell lines separately according to the recommended medium of it. In addition, we added 10% (v/v) fetal bovine serum (FBS, Gibco, USA) to the medium as well as diluted to a concentration of 100 U/mL penicillin and 100 *μ*g/ml streptomycin. All cells were resuscitated on a sterile ultraclean table following aseptic procedures and then incubated in an incubator at 37°C in 5% CO_2_. In addition, we also performed hypoxic modeling of the cells and set the incubator conditions at 1% O_2_, 5% CO_2_, and 94% N_2_.

### 2.2. Real-Time Quantitative (RT-qPCR)

Total RNA was extracted with TRIzol reagent (Invitrogen, USA) according to the instructions. Then, we used the Tall Capacity RNA-to-cDNA Pack (Connected Biosystems) kit and mixed the extracted RNA diluted to the appropriate concentration according to the instructions and put it into the machine for testing. QPCR was performed by utilizing the taking after preliminaries: WTAP (forward) 5′-TTGTAATGCGACTAGCAACCAA-3′ and (reverse) 5′- GCTGGGTCTACCATTGTTGATCT-3′; and 18S rRNA, 5′-CGG CGACGA CCC ATT CGA AC-3′ and 5′-GAA TCG AAC CCT GAT TCC CCGTC-3′. DGCR8. (forward) 5′-GCCTCCTCATAGACCCGAACT-3′ and (reverse) 5′- CGGTAAAGCTCACGCTAATCTT-3′; miR-200 5′-TAATACTGCCGGGTAATGATGGA-3′; U6: CTCGCTTCGGCAGCACA.

### 2.3. CCK8 Assay

After stable transfection of cells with interference, the control and sh-WTAP cell groups were diluted and inoculated in 96-well plates at a density of 2000 cells per well with 100 *μ*L of total medium. Each of these cells was grown in five wells of the plate. 10 *μ*L Cell Tallying Kit-8 (CCK-8, WST-8, Dojindo, Japan) arrangement was included in each well after 0 h, 24 h, 48 h, 72 h, and 96 h, separately. In practical cells, CCK8 was metabolized to create a colorimetric color identified at 450 nm employing a microplate peruser. The experiment was rehashed three times.

### 2.4. Transwell Assay

To analyze the migratory capacity of transfected cells, 5 × 10^4^ cells were added to the upper chamber of 8 *μ*m transwell inserts (BD Biosciences, USA) in the serum-free medium. Then, 200 *μ*L of FBS-containing medium was added to the lower chamber. After incubation in the incubator for 24 h, the chambers were removed, washed and dried repeatedly with PBS solution, and stained with 0.1% crystal violet. Next, the stained cells were observed under an inverted microscope. For the transwell invasion assay, all steps were the same as for the migration assay, except that the upper chamber was coated by the Matrigel (BD Biosciences).

### 2.5. Wound Healing Assay

Wound mending relocation measures were performed as depicted. Briefly, cells were seeded in a six-well chamber slide at a thickness of 5 × 10^3^ cells/well in Dulbecco's altered Eagle's medium. Scratches were performed within the middle of falls employing a sterile 10 *μ*L pipette tip. After brooding for 48 hours, photos were taken to gauge the closure of the crevice.

### 2.6. RNA Immunoprecipitation

The procedure was performed according to the instructions issued by RIP kit (Abcam, UK). First, we collected the A2780 cells to be used and treated them with formaldehyde to form cross-linked protein-RNA complexes, then centrifuged the cells by centrifugation, and resuspended the nuclei in freshly prepared RIP buffer, followed by chromatin fragmentation of the buffer. After immunoprecipitation and purification, the obtained RNA was reverse transcribed into cDNA and further sequenced for analysis.

### 2.7. Clinical Samples

The human ovary tissue microarray contained 276 cases of OC. These tissues were gotten from the obstetrics and gynecology office of the First Affiliated Hospital of Wannan Medical College. None of them had gotten chemotherapy, radiotherapy, and other related antitumor treatments. All human ovary tissues were gotten with educated assent, and the moral audit committee affirmed all conventions of the World Wellbeing Organization Collaborating Center for Investigate in Human Generation.

### 2.8. Immunohistochemical Staining

OC tumor tissue was carefully cut into 4 *μ*m thick sections and then immunostained with primary antibody (1 : 200, Rabbit, Abcam) of WTAP. All experimental steps of this experiment were performed in strict accordance with the standard experimental procedures. First, the cut tissues are sealed, fixed and dewaxed, followed by antigen repair of the sections, followed by background closure to minimize the weakness of the nonspecific signal of the sections, followed by detection with antibodies to WTAP, and finally photographed and imaged on the microscope. And all sections were scored based on the percentage and intensity of positively stained cells: 0-5%, 0; 6-35%, 1; 36-70%, 2; and more than 70%, 3. All OC tissue sections were then divided into low expression group or high expression group according to the scoring, where low expression group: score 0-1; high expression group: score 2-3 [29].

### 2.9. Animal Studies

The animal studies in this project were approved by the Committee of Scientific Research and New Technology IRB of Wannan Medical College Yijishan Hospital (2019-31). Female thymus-free BALB/c nude mice, mainly 5 weeks in size, were used in this experiment. Prescribed specific OC cell lines were injected into these mice by different methods, subcutaneous tumor growth assay, liver metastasis model, and tail vein injection model experiments.

### 2.10. Statistical Analysis

All data was reported as mean ± standard error (SEM) of at least three independent experimental results. The Student *t*-test was used for continuous variables. And the Kaplan-Meier analysis and log-rank test were used to calculate whether the survival rate of patients was related to the expression of WTAP. In the process of statistics, we also use the statistical method of Pearson's correlation coefficient. Throughout the process, statistical analysis was performed using the SPSS software package (version 21.0; IBM SPSS, Chicago, IL). *P* < 0.05 was considered statistically significant.

## 3. Results

### 3.1. HIF-1*α* Promotes m6A Methylase WTAP and m6A RNA Methylation in OC Cells under a Hypoxic Environment

**Figure 1:**
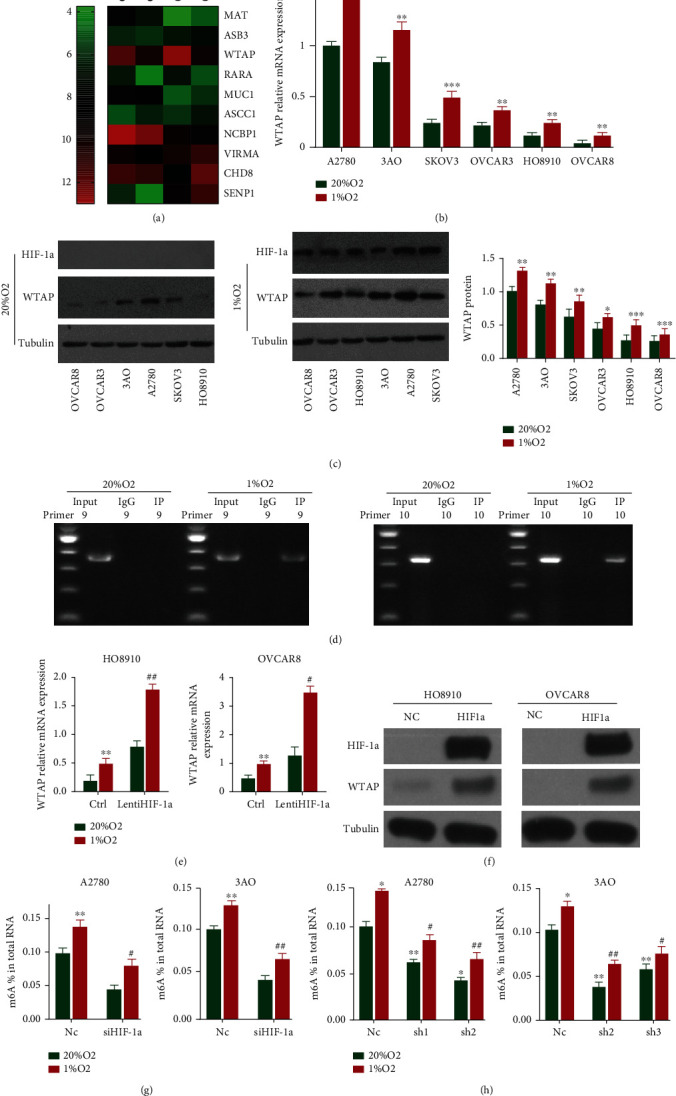
Hypoxia-induced and HIF-1*α*-dependent WTAP expression mediates an alteration in total RNA m6A levels. (a) WTAP were remarkably upregulated under hypoxic microenvironment of 1% O2 condition compared to 20% O_2_ condition in two ovarian cancer cell lines (3AO and A2780) through analysis of whole genome expression profile. (b) Six human ovarian cancer cell lines (A2780, 3AO, SKOV3, OVCAR3, HO8910, and OVCAR8) were exposed to 20% or 1% O_2_ for 24 h, and WTAP mRNA expression was determined by RT-qPCR relative to 18S rRNA and normalized to the mean value for A2780 cells at 20% O_2_ (^∗∗^*P* < 0.01 and ^∗∗∗^*P* < 0.001). (c) OC cells were exposed to 20% and 1% O_2_ for 48 h, Western blot experiments were performed to analyze HIF-1*α* and WTAP protein expression. Tubulin was detected as a loading control. ( ^∗∗^*P* < 0.01, and ^∗∗∗^*P* < 0.001). (d) CHIP-PCR experiment was applied and the results demonstrated that HIF-1*α* which acted as a transcription factor could bind to the region corresponding to primer 9 and 10 in the promoter region of WTAP. (e) HO8910 and OVCAR8 Ctrl and LentiHIF-1*α* subclones were exposed to 20% or 1% O_2_ for 24 h. WTAP mRNA expression levels were determined as a percentage of all adenosine residues in RNA (^∗∗^*P* < 0.01 vs. 20% O_2_; ^#^*P* < 0.05 and ^##^*P* < 0.01 vs. 1% O_2_ Ctrl). (f) Western blot experiments were performed to detect the expression levels of HIF-1*α* and WTAP. (g) A2780 and 3AO Nc and siHIF-1*α* subclones were exposed to 20% or 1% O_2_ for 24 h. m6A levels were determined as a percentage of all adenosine residues in RNA (^∗∗^*P* < 0.01 vs. 20% O_2_; ^#^*P* < 0.05 and ^##^*P* < 0.01 vs. 1% O_2_ NC). (h) A2780 and 3AO Nc and WTAP knockdown (sh1 sh2 in A2780 and sh2 sh3 in 3AO) subclones were exposed to 20% or 1% O_2_ for 24 h. m6A content was determined as a percentage of all adenosine residues (^∗^*P* < 0.05 and ^∗∗^*P* < 0.01 vs. 20% O_2_; ^#^*P* < 0.05 and ^##^*P* < 0.01 vs. 1% O_2_ NC).

Two OC cell lines (3AO and A2780) were revealed to 20% or 1% O_2_ for 24 h, and a gene-wide expression profiling chip was made. After analysis by hot map, we found that m6A methylase WTAP was significantly upregulated in 1% O_2_ condition (Figure 1(a)). And WTAP mRNA levels were upregulated under hypoxic conditions (1% O_2_) in the six OC cell lines (Figure 1(b)). The western blot investigation showed that HIF-1*α* and WTAP expression were identified in OC cell lines uncovered to 1% O_2_ and reliable with mRNA expression. The protein expression level of WTAP was also essentially upregulated in six OC cell lines activated by hypoxia (Figure 1(c)). To determine whether hypoxia-induced WTAP expression depended on HIF-1*α*, we further verified the regulatory relationship between HIF-1*α* and WTAP using the CHIP-PCR technique. The results showed that beneath hypoxia (1% O_2_), HIF-1*α* might tie to the locale compared to preliminary 9 and 10 within the promoter locale of WTAP, proposing that its regulatory site is found within the 600 bp region upstream of the ORF locale of human WTAP gene (Figure 1(d)). This illustrated that there exists a coordinated administrative relationship between HIF-1*α* and WTAP.

Besides, we detected the expression of WTAP in OVCAR8 and HO8910 cells after the stable overexpression of HIF-1*α*. The results proved that the expression of WTAP increased consistently with the increase of HIF-1*α* expression both at mRNA and protein level (Figures 1(e) and 1(f)). We further explored whether the increased expression of WTAP in cells under hypoxic conditions could change the m6A methylation level in whole-cell RNA. We firstly knockdown HIF-1*α* in A2780 and 3AO cells, separately, and found that the m6A level in RNA diminished significantly, revealing that the downregulation of HIF-1*α*-initiated WTAP expression seems to annul the m6A methylation impacts (Figure 1(g)). Then, we generated subclones of A2780and 3AO that were stably transfected with two different shRNAs targeting WTAP. The consequence showed m6A level in RNA in both cell lines decreased significantly (Figure 1(h)), indicating that decreased WTAP expression could inhibit the m6A methylation effects.

### 3.2. WTAP Protein Is Upregulated in OC Tissues and Closely Correlates with Clinicopathology

**Figure 2:**
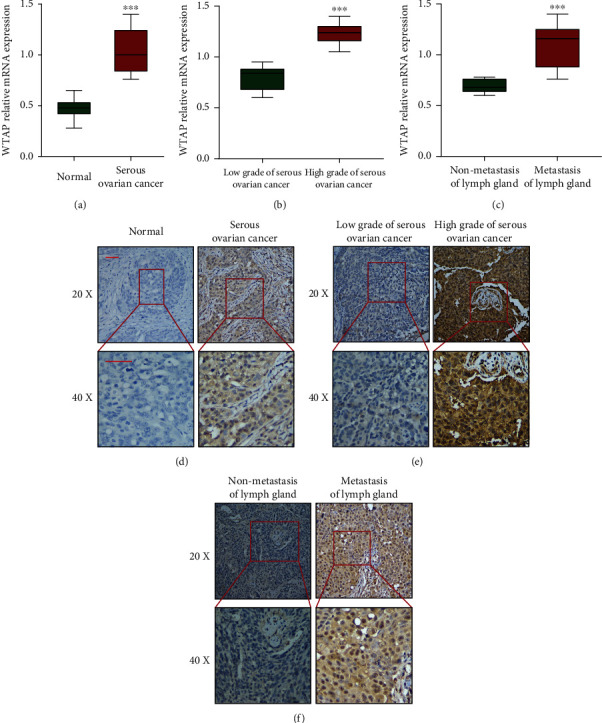
The expression of WTAP is significantly upregulated in ovarian cancer tissue and closely correlated with clinicopathological parameter. (a) The mRNA expression of WTAP was upregulated in serous OC tissues compared with normal tissues. (b and c) WTAP expression was relatively higher in high grade OC tissues and those with metastasis of the lymph gland. ^∗∗∗^*P* < 0.001. (d) Comparisons of WTAP expression in tissues revealed by IHC analysis in normal and serous OC tissues. (e) Comparisons of WTAP expression in tissues revealed by IHC analysis, low grade and high grade of serous OC tissues. (f) Comparisons of WTAP expression in tissues revealed by IHC analysis, nonmetastasis and metastasis of lymph gland tissues. The positive staining of WTAP was presented in brown color (distributed in both nucleus and cytoplasm), and the cell nuclei were counterstained with hematoxylin. Original magnification, 20x (left panels) and 40x (right panels). Scale bars = 10 *μ*m.

To assess the expression status of WTAP in human OC tissues, we identified the mRNA expression of WTAP in 30 matched serous OC and adjoining ordinary tissues. The expression of WTAP was essentially lifted in cancer tissues compared with typical tissues (Figure 2(a)). And WTAP emerged a higher expression tendency in high-grade OC tissues and those with metastasis of the lymph gland (Figures 2(b) and 2(c)). To obtain a further understanding of the clinical significance of WTAP in OC, we then performed an immunohistochemical analysis of a tissue microarray that contained 276 serous OC tissue samples and 86 normal ovarian tissues. Immunohistochemical staining showed that the expression of WTAP in OC tissue was altogether higher than that in the ordinary ovarian tissue (Figure 2(d)). High-grade OC tissues and tissues with metastasis of the lymph gland emerged a significantly stronger expression than low-grade OC tissues or nonmetastasis of the lymph gland (Figures 2(e) and 2(f)). To assist in exploring the clinical centrality of WTAP expression in OC, we studied the relationship between the WTAP expression status and clinicopathological characteristics of 276 OC patients separated into the high expression (*n* = 155) and low expression group (*n* = 121). The results demonstrated that the WTAP expression was surprisingly related to histologic subgroups (*P* = 0.013), FIGO organize (*P* = 0.001), and lymph hub status (*P* = 0.02) (Table 1). These results proved that the WTAP expression was emphatically related to tumor movement in OC.

### 3.3. High Expression of WTAP Was Unequivocally Related to Destitute Prognostic Components in Patients with OC

**Figure 3:**
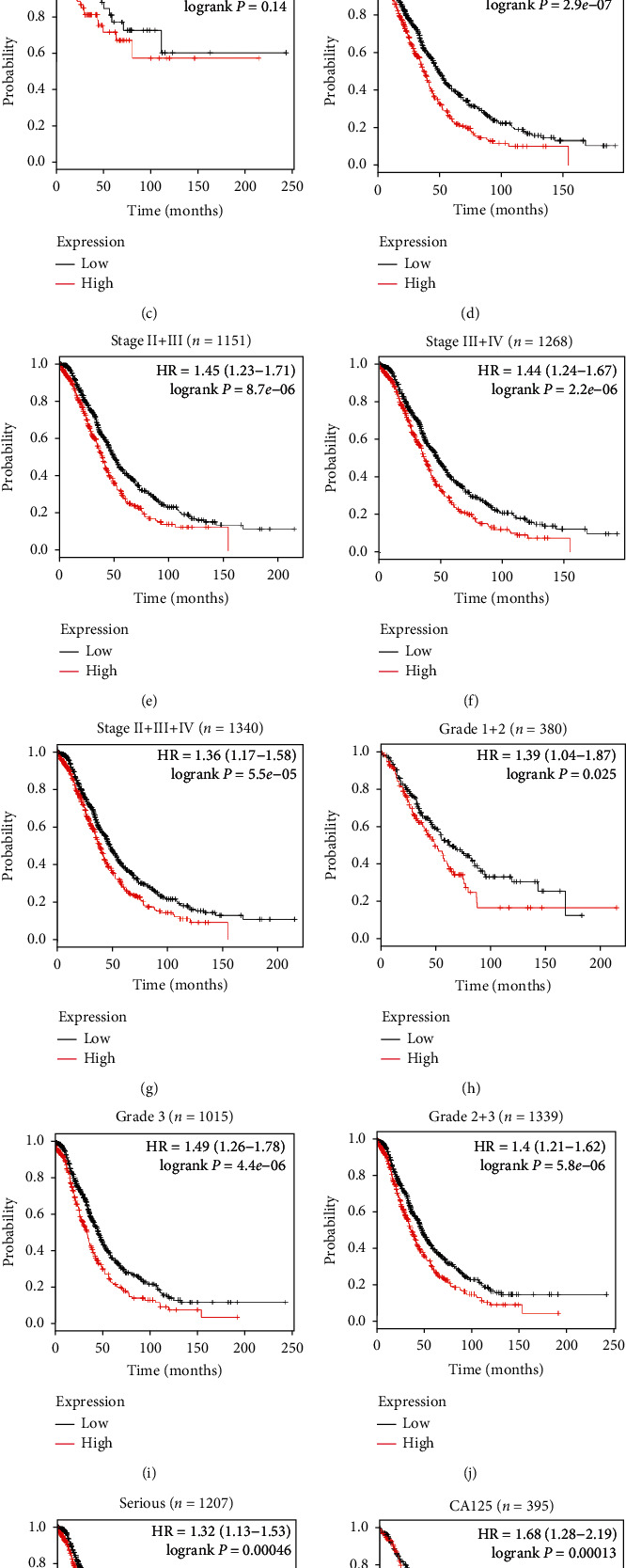
The Kaplan-Meier analysis of overall survival in OC patients based on K-M plotter dataset. (a) The expression of WTAP was negatively correlated with overall survival (OS) in OC patients; (b–k) Correlation between WTAP expression and overall survival was independent of clinical stage (b–g), grade (h–j), and tumor type (k). (l) High expression of WTAP predicted poor prognosis in OC patients regardless of CA125 level. *P* values were calculated by log-rank test.

To decide the clinical importance of WTAP, its prognostic esteem in OC was inspected by analyzing survival information of more than 1000 OC patients from the KM plotter dataset (http://kmplot.com/analysis). It was found that the tall expression of WTAP was altogether contrarily connected with the overall survival of OC patients in KM plotter (*n* = 1657, *P* < 0.001, Figure 3(a)), showing that patients with higher WTAP levels would have an essentially poorer forecast than those with lower WTAP levels. In addition, expanded expression of WTAP was strikingly related to destitute overall survival of patients, notwithstanding of the FIGO organize (Figures 3(b)–3(g)), grade (Figures 3(h)–3(j)), tumor type (Figure 3(k)), and CA125 level (Figure 3(l)).

### 3.4. WTAP Knockdown Decreased the Proliferation Capacity *In Vitro* and *In Vivo*

Since OC showed more extensive WTAP expression, we further examined WTAP knockdown influences on OC proliferation. We performed stable interference transfection with shRNA-WTAP plasmids in WTAP relatively high expression cell lines A2780 and 3AO, with effective fragments sh1 and sh2 in A2780 cells and sh2 and sh3 in 3AO cells. In addition, we also transfected the corresponding cells with mock vectors labeled with Nc. The silencing effects of the shRNAs in these two cell lines were validated by RT-qPCR (Supplementary figure 1A). To investigate the impact of WTAP on OC cell lines, we performed tests utilizing steady cell lines of A2780-WTAP and 3AO-WTAP and utilized CCK8 and plate colony arrangement measures to identify changes within the expansion of OC cell lines after WTAP impedances. Both tests showed that knockdown of WTAP expression essentially restrained the expansion of A2780 and 3AO *in vitro* (Supplementary figure 1B–C).

Moreover, the inhibitory effect of silencing WTAP on proliferation was verified *in vivo* by subcutaneously inoculating A2780-sh2 and Nc cells into nude mice. Six weeks later, tumors derived from the sh2 cells were significantly smaller than those derived from Nc cells (S1D). The tumor growth rate was remarkably suppressed after WTAP silencing, and the tumor weight was substantially smaller in A2780-sh2 nude mice (Supplementary figure 1E–1F). Generally, all the above exploratory suggested that knockdown of WTAP in OC cell lines significantly repressed the proliferation capacity of OC cells *in vitro* and essentially diminished the capacity of OC cells to advance tumorigenesis *in vivo*.

### 3.5. WTAP Knockdown Diminished the Migration Capacity *In Vitro* and *In Vivo*

**Figure 4:**
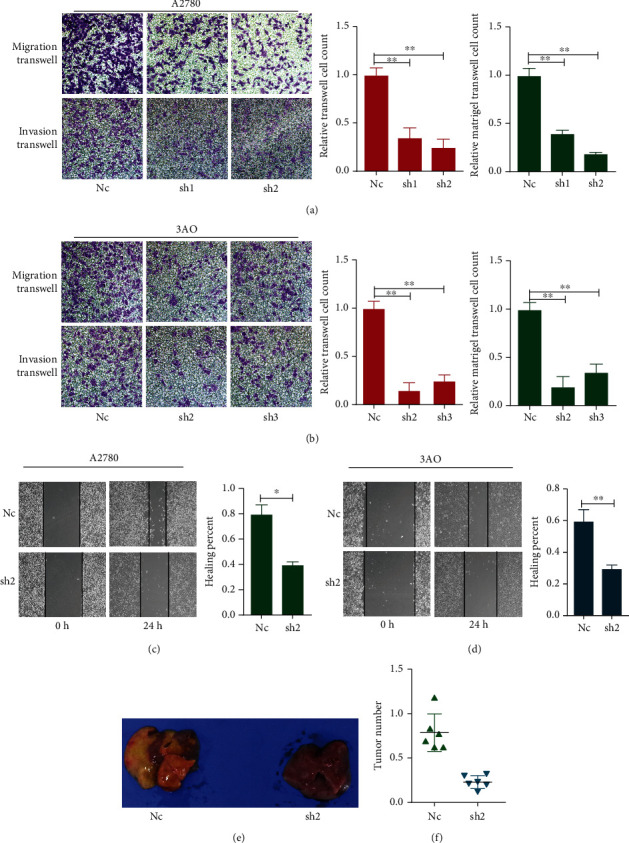
Silencing of WTAP reduces the migration and invasive capacity of OC cells *in vitro* and metastasis *in vivo*. (a and b) Representative migration and invasion images of WTAP silenced and Nc cells in A2780 and 3AO. Original magnification: 200x. Calculations of cell number were performed with three randomly selected fields. (c and d) Representative wound healing images of A2780 and 3AO at 0 and 24 h, respectively, the cell boundary was marked by the dotted black line. ^∗^*P* < 0.05, ^∗∗^*P* < 0.01. (e) Pulmonary metastasis models were established and typical diagram was shown. (f) Statistical analysis of numbers of pulmonary metastatic nodules.

In order to understand whether WTAP can have an effect on the migration and invasion ability of OC, we therefore used the transwell assay and wound healing assay to test one by one. The results showed knockdown of WTAP notably blocked the migration and invasion of A2780 and 3AO cells compared with the Nc cells (Figures 4(a)–4(d)). To further in depth verify the effect of WTAP on biological functions *in vivo*, we injected A2780-Nc or A2780-sh2 cells into nude mice and examined their liver tumorigenesis. After six weeks of cautious raising, we executed mice treated with uncommon cancer cells utilizing the cervical dislocation, and their livers were dismembered and inspected to watch the metastasis of the tumors. The results suggested that knockdown of WTAP essentially repressed the metastatic capacity of OC cell lines *in vivo* (Figure 4(e)). And the number of liver metastatic nodules was significantly reduced in mice inoculated with A2780-sh2 cells compared with mice inoculated with A2780-Nc cells (Figure 4(f)).

### 3.6. Overexpression of WTAP Enhanced the Proliferation, Migration, and Invasion Abilities of OC Cells

In order to further understand the biological function of WTAP in OC, H8901 and OVCAR8 (OC cell lines with relatively low WTAP expression) were specifically processed to perform biological function. Both cell lines were transfected with stable overexpression of Lenti-WTAP and plasmids labeled with over mimic vectors in cell lines with low WTAP expression. And RT-qPCR (Supplementary figure 2A) was used to verify the overexpression efficiency of stable overexpression. Next, we performed CCK8 assays using the constructed stably transfected cell lines and then examined the proliferation of cells after transfection of Lenti-WTAP cells and Nc cells. The results demonstrated that overexpression of WTAP remarkably enhanced the proliferative capacity of both H8901 and OVCAR8 cell lines (Supplementary figure 2B). In addition, we also performed a transwell assay to detect the invasive migration ability of WTAP overexpressed cell lines using the aforementioned constructs, and the results showed that WTAP overexpression significantly promoted the migration and invasive ability of H8901 and OVCAR cells (Supplementary figure 3C–F). In addition, we further verified the effect of WTAP on the migratory ability of cells using the scratch wound healing assay (Supplementary figure 2G–H), and the results were found to be consistent with the above experimental results, i.e., it was significantly stronger in WTAP overexpressed cells than in the control group.

### 3.7. WTAP-Dependent m6A Methylation Regulates the Processing of miR-200 Maturation by Targeting DGCR8

**Figure 5:**
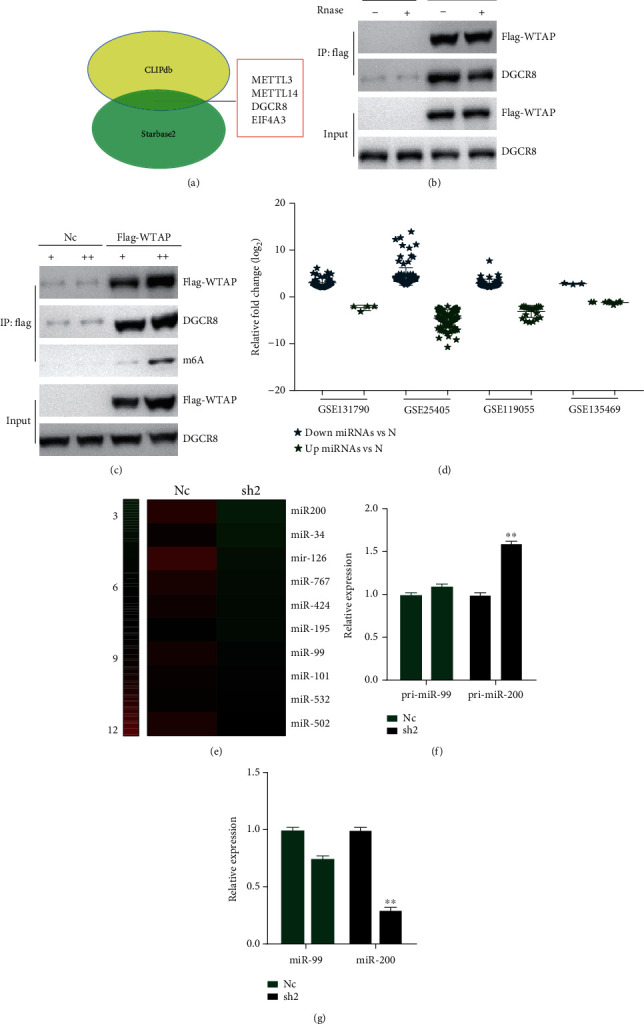
WTAP-dependent m6A demethylation regulates the processing of miR-200 via DGCR8. (a) CLIPdb and starBase2 databases were used to predict the interaction proteins of WTAP. (B) Coimmunoprecipitation of the WTAP-interacting protein DGCR8. A2780 cells were crosslinked before the immunoprecipitation. WTAP gene was tagged by flag in advance. Ribonuclease was applied as indicated. Immunoglobulin G antibody was used as negative control for the experiment. (c) Immunoprecipitation of DGCR8, WTAP, and associated RNA from control cells or cells overexpressing WTAP (+ stands for control, ++ represents over expressed WTAP flag). Western blot and immunoblot experiments were done using the antibodies as depicted. (d) Downregulated and upregulated miRNAs were analyzed and selected in OC tissues compared to adjacent normal tissues from Gene Expression Omnibus microarray profiles (GSE131790, GSE25405, GSE119055, and GSE135469). (e) Gene-wide expression profiling chip was fabricated in A2780-Nc and A2780-sh2 cells to screen differentially expressed miRNAs after WTAP knockdown. (f) Pri-miR-99 and pri-miR-200 were detected by RT-qPCR upon WTAP silencing in A2780 cells. (g) MiR-99 and miR-200 expressions were detected by RT-qPCR upon WTAP silencing in A2780 cells. Annotation: RNase = ribonuclease; IP = immunoprecipitation; IgG = immunoglobulin G; oe = overexpressing).

Studies discovered that m6A methylase METTL14 could regulate pri-miRNA processing by directly targeting DGCR8 in a way dependent on METTL14/m6A in hepatocellular carcinoma [30]. We found that most of these consider emphasizing the critical part of m6A methylation adjustment in pri-miRNA preparing. Subsequently, in our consideration, we hypothesized that WTAP (another basic m6A methylester) moreover has a vital part in miRNA arrangement, particularly whether it is straightforwardly included within the official of DGCR8 to pri-miRNA in OC. We first predicted the interaction protein of WTAP in several OC databases (CLIPdb and starBase2). After the intersection, what was shocking was that, besides METTL3/METTL14, DGCR8 was a very likely interacting protein of WTAP (Figure 5(a)). Then, we performed immunoprecipitation assays and found that WTAP could significantly bind to DGCR8, and further RNase treatment of the cells revealed that the binding of WTAP and DGCR8 was significantly weaker than before in the immunoprecipitation assay (Figure 5(b)). Then, we reasonably proposed that WTAP might be similar to METTL3/METTL14, which is a methylation RNA enzyme that affects miRNA after binding to DGCR8. Further experiments revealed that we also observed a significant increase in DGCR8-bound methylated RNA in WTAP overexpressing cells (Figure 5(c)). All the above results suggest that WTAP may affect the formation of pri-miRNA by manipulating DGCR8 and thus affecting the recognition and binding of DGCR8 to pri-miRNA.

Based on the above experimental results, we next wanted to further verify whether WTAP could regulate miRNA expression by regulating DGCR8. We used microarray databases of OC for bioinformatic prediction, which were collected from the National Center for Biotechnology Information's comprehensive gene expression database (GSE131790, GSE25405, GSE119055 GSE135469). We analyzed the microarray data to acquire differently expressed miRNAs between OC or normal tissues (Figure 5(d)). In our experiments, given the significantly increased levels of m6A methylation in OC, we preferentially selected significantly upregulated miRNAs as candidates for WTAP regulation. Meanwhile, to further screen out WTAP-regulated miRNAs more accurately, we performed whole gene expression profiling microarray sequencing experiments using A2780-Nc and A2780-sh2 cell lines, which were analyzed by hot map after sequencing. Interestingly, we found that several miRNAs interfered with WTAP in the sequencing experiments had significant downregulation of multiple miRNAs (Figure 5(e)). Finally, we took the intersection of the above database screened miRNAs and the downregulated miRNAs obtained by sequencing and finally found that miR-200 and miR-99 are common miRNAs. Next, we also examined the expression of the two selected pri-miRNAs and their corresponding miRNAs in WTAP-interfered cell lines, and the results suggested that only the expression of pri-miR-200 increased significantly with the decrease of WTAP in the cell lines (Figure 5(f)). In contrast, the expression of the corresponding miR-200 was significantly decreased after silencing of WTAP expression (Figure 5(g)).

**Figure 6:**
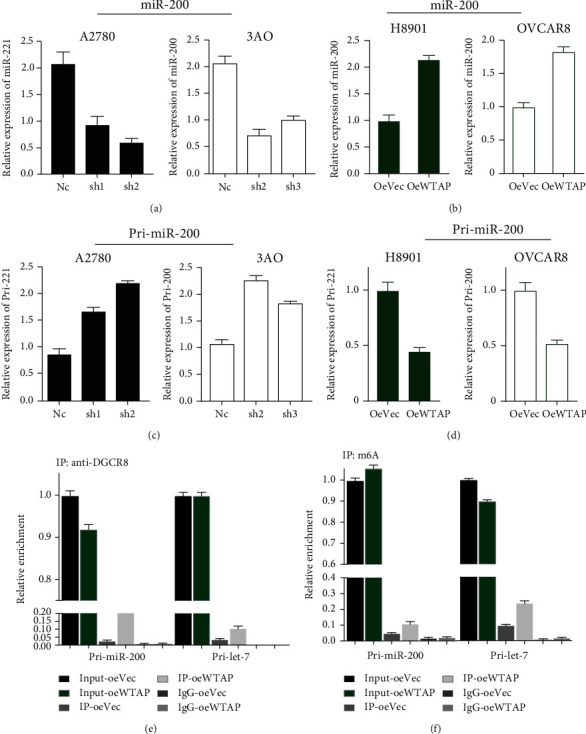
WTAP-dependent m6A demethylation regulates the processing of miR-200 via DGCR8. (a) MiR-200 was detected using RT-qPCR upon WTAP depletion or overexpression in A2780 and 3AO cells. (b) MiR-200 was quantified using RT-qPCR upon WTAP overexpression in H8901 and OVCAR8 cells. (c and d) pri-miR200 was detected by RT-qPCR upon WTAP depletion or overexpression in A2780/3AO and H8901/OVCAR8 cells. (e) Immunoprecipitation of DGCR8-associated RNA from control or WTAP-overexpressing cells followed by RT-qPCR to detect pri-miRNA binding to DGCR8. Pri-let-7e was used as a positive control. Three independent experiments were performed. (f) Immunoprecipitation of m6A modified RNA in control or WTAP-overexpressing cells followed by RT-qPCR to estimate the m6A modification levels of pri-miRNAs. IgG = immunoglobulin G; IP = immunoprecipitation; oe = overexpressing).

Then, we again utilized the constructed sh-WTAP or Lenti-WTAP cell lines and assayed this series of cell lines to examine the expression of pri-miR-200 and miR-200 therein. The results showed that in WTAP-silenced cells (A2780 and 3AO), the expression of mature miR200 was significantly reduced, while in contrast, in WTAP-overexpressing cell lines (H8901 and OVCAR8), the expression of mature miR200 was significantly increased, as follows Figures 6(a) and 6(b). In contrast, in interfering WTAP, the expression of pri-miR-200 was in contrast to the expression of miR-200, and its expression was consistent with WTAP expression, i.e., decreased with the knockdown in WTAP. Similarly, in cell lines overexpressing WTAP, its expression was consistent with WTAP expression, i.e., it was upregulated with increasing low in WTAP (Figures 6(c) and 6(d)). In addition, we performed immunoprecipitation experiments using lenti-ctrl with Lenti-WTAP cell lines and further detected using RT-qPCR that in OC cell lines overexpressing WTAP, the increase in WTAP expression was followed by a corresponding increase in DGCR8 bound to it, and the level of pri-miR-200 bound to DGCR8 increased accordingly (Figure 6(e)). In addition, we again performed RNA immunoprecipitation experiments using our control and WTAP overexpressing stable cell lines, and the results suggested that overexpression of WTAP significantly increased the expression of m6A-modified pri-miR-200 (Figure 6(f)). In conclusion, all the above results indicated that WTAP could significantly enhance the recognition of pri-miR-200 by DGCR8, as well as the subsequent WTAP could enhance m6A methylation to promote pri-miR processing into mature miRNA.

### 3.8. Overexpression of miR-200 Altogether Turned Around the Expansion and Movement Capacity of WTAP-Promoted Cancer Cells in OC

To begin with, we inspected the expression of miR-200 in OC, and the results proposed that the expression of miR-200 in OC tissues was altogether higher than that in typical tissues (Supplementary figure 3A). When we transfect miR-200 mimics in WTAP knockdown cells, we found that in the constructed cells stably transfected with WTAP knockdown A2780 and 3AO, retransfection with miR-200 mimics significantly partially increased the proliferation of OC and reversed the effect of WTAP reduction on cell proliferation in cells (Supplementary figure 3B). Accordingly, miR-200 mimics could increase the cell migration capacity induced by WTAP inhibition (Supplementary figure 3C–3D). Taken together, these results demonstrate a critical role of OC cell-intrinsic WTAP in promoting cancer cell progression by regulating DGCR8-miR-200 (Supplementary figure 4).

### 3.9. Upregulation of WTAP Essentially Advances the Warburg Impact on OC Cells

**Figure 7:**
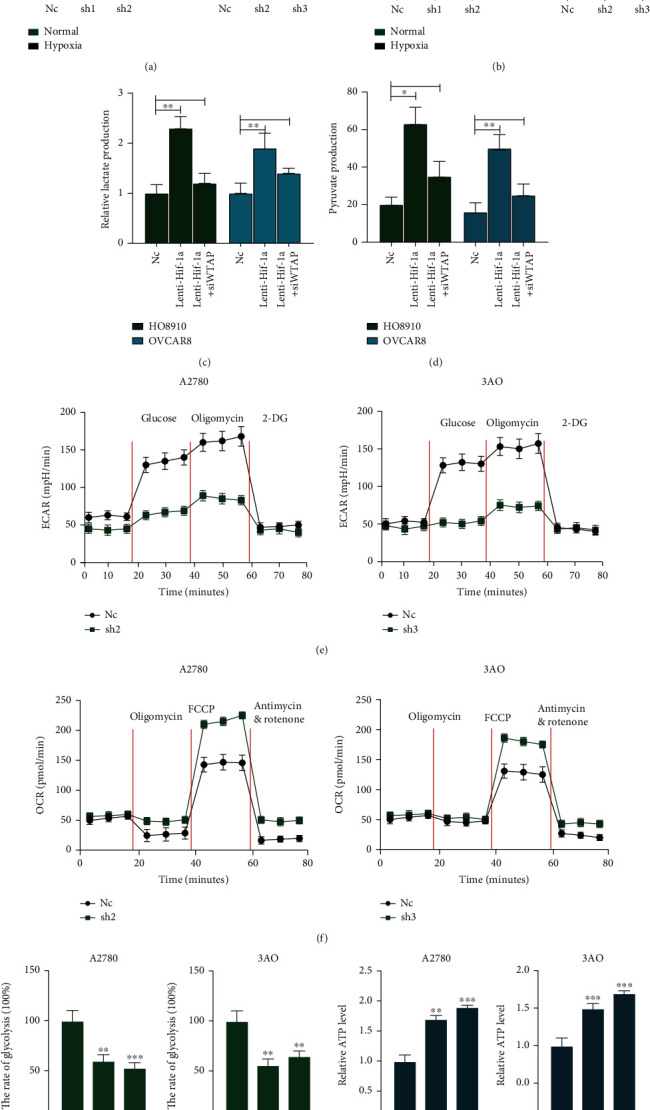
WTAP promotes the Warburg effect in OC cells. (a and b) Alterations in lactate production (a) and pyruvate production (b) levels were analyzed after transfection of A2780 and 3AO cells with sh-NC, sh-WTAP in normal and hypoxia environment, ^∗∗^*P* < 0.01 vs. NC group; ^##^*P* < 0.01 vs. Normal group. (c and d) Alterations in lactate production (c) and pyruvate production (d) levels were analyzed after transfection of HO8910 and OVCAR8 cells with NC, Lenti HIF-1*α*, SI-WTAP reversing this effect. ^∗^*P* < 0.05 and ^∗∗^*P* < 0.01. (e) The extracellular acidification rate was analyzed after transfection of A2780 and 3AO cells with sh-NC, sh-WTAP. (f) The oxygen consumption rate was analyzed after transfection of A2780 and 3AO cells with sh-NC, sh-WTAP. (g and h) The glycolysis rate (g) and ATP level (h) were detected after transfection of A2780 and 3AO cells with sh-NC, sh-WTAP.

Based on the above findings, we studied whether WTAP expression affected the Warburg effect by measuring pyruvate and lactic acid expression levels and the glycolysis rate in OC cells. Statistical analysis demonstrated that knockdown of WTAP significantly reduced lactate production and pyruvate expression in A2780 and 3AO cells. In addition, we found that lactate production and pyruvate expression were upregulated in WTAP knockdown cells cultured under hypoxia (Figures 7(a) and 7(b)). To further verify that hypoxia affects the glycolytic process by regulating WTAP through HIF-1*α*, we examined the expression of lactate and propionate after overexpression of HIF-1*α* in HO8910 and OVCAR8 cell lines, and the results suggested that the expression of lactate and propionate increased significantly after overexpression through HIF-1*α*, and this effect was reversed after interference with WTAP in the constructed cell lines, and all the above results suggested that HIF-1*α* affects the process by regulating WTAP (Figures 7(c) and 7(d)). In addition, we analyzed the extracellular acidification rate (ECAR) in WTAP-disrupted stable-transformed OC cell lines using the XF24 cell efflux analyzer (Seahorse) and showed that disrupting WTAP expression significantly reduced the ECAR of OC cells compared to control OC cell lines (Figure 7(e)). We also examined the oxygen consumption rate (OCR, a marker of OXPHOS) in the same cell lines, and the results also suggested that the OCR capacity of OC cells was significantly enhanced after knocking down WTAP compared to control cells (Figure 7(f)). The results confirmed that WTAP knockdown could reduce OC cells' glycolysis rate and glycolysis ability (Figure 7(g)). Besides, the knockdown of WTAP in A780 and 3AO cells also increased the formation of ATP produced by OXPHOS (Figure 7(h)). The above results demonstrated that the Warburg effect was significantly enhanced in OC cells after knockdown of WTAP.

### 3.10. Knockdown of HK2 Significantly Blocked the Antitumor Effect of miR-200 in OC Cells

**Figure 8:**
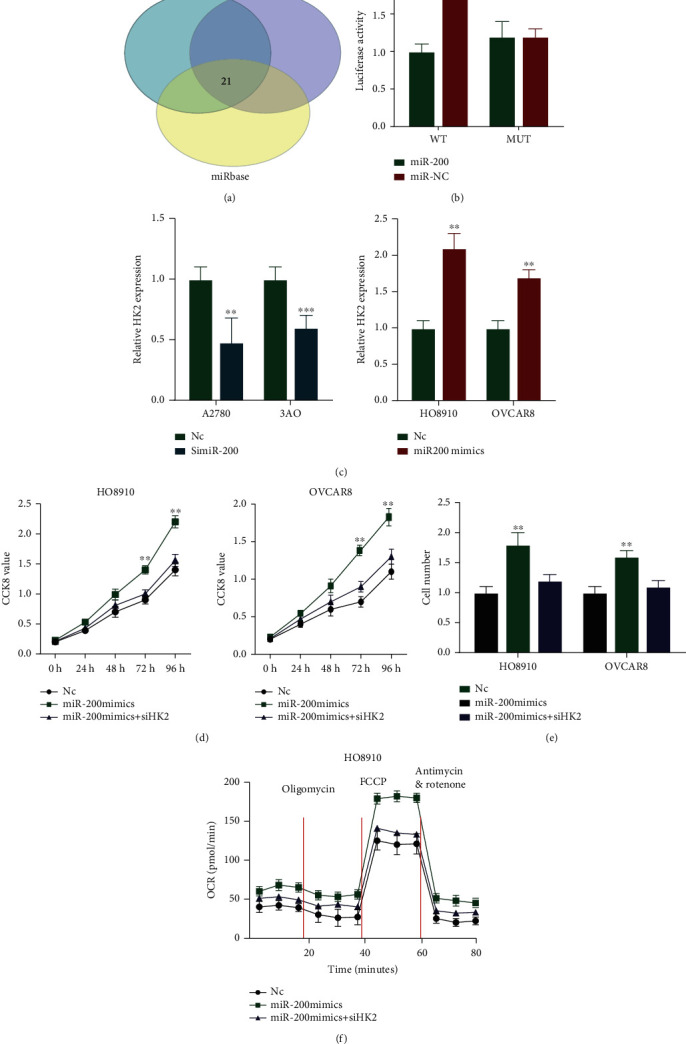
HK2 was regulated by miR-200 and silencing of HK2 abolishes the effects of miR-200 in OC cell lines. (a) Analysis of TargetScan, Pictar, and miRBase datasets identified 21 genes as potential target of miR-200. (b) Assay of dual-luciferase reporter was performed to explore the binding relationship between miR-200 and HK2 in 293T cells. (c) The expression of HK2 in OC cells with si-miR-200. (d) Effect of reintroduction of HK2 on miR-200-mimics cell proliferation by CCK-8 assay; (e) Effect of reintroduction of HK2 on miR-200-mimics invasion ability by transwell assay; (f) The oxygen consumption rate was analyzed after transfection of OC cells. ^∗∗^*P* < 0.01.

Hexokinase (HK) is a classical enzyme that catalyzes the phosphorylation of hexose. Existing studies have shown that HK2 has the most significant correlation with tumors among the four H.K. isoenzymes [31]. However, whether HK2 is regulated in relation to miR-200 in OC has not been reported in the literature. This study found that miR-200 can regulate HK2 by using databases such as miRBase, mirwalk, and Pictar (Figure 8(a)). Therefore, we used relevant experiments to further verify whether the database predictions existed. First, we analyzed whether HK2 was regulated by miR-200 using a luciferase reporter gene assay, and the results clearly suggested that miR-200 positively regulated the expression of this key glycolytic enzyme HK2 (Figure 8(b)). In addition, after knocking down miR-200 expression in OC cell lines, the expression of HK2 decreased. And overexpression of miR-200 significantly upregulated the HK2 expression (Figure 8(c)). To further confirm the interaction between miR-200 and HK2, further experiments were carried out. Firstly, the CCK8 investigation showed that the cell proliferation capacity was enhanced after overexpression of miR-200 while knocking down of HK2 could reverse its proliferation ability (Figure 8(d)). Secondly, we found that the cells' migration capacity was enhanced after overexpression of miR-200 while knocking down of HK2 could reverse the effect (Figure 8(e)). Finally, we also measured the oxygen consumption rate (OCR, marker of OXPHOS) and found that knocking down of miR-200 could enhance the OCR ability of OC cells while knocking down of HK2 could reverse its OCR ability (Figure 8(f)). In summary, all our experimental results indicate that miR-200 can positively regulate the expression of key no HK2 in glycolysis, which in turn significantly affects the Warburg effect in OC cells and promotes tumorigenesis.

## 4. Discussion

Increasingly, it has appeared that RNA m6A alteration plays an awfully critical part in the event and advancement of harmful tumors [32]. It is now well established that dynamically balanced m6A modifications regulated by m6A methyltransferases and demethylases are involved in a wide range of cellular biological processes, such as variable splicing of RNA, protein translation, and regulation of stem cell pluripotency [17, 33]. Because of its vital biological role, increasing studies have focused on the part of m6A modification in human cancers, including OC [34]. WTAP, which has been shown to be a key factor in m6A methylation, plays a critical role in the methyltransferase complex because it not only mediates m6A methylation of RNA [32, 35]. Various literature has confirmed that WTAP, which acts as a critical m6A methylase, profoundly contributes to various cancers' pathogeneses. For example, the available literature reports that in hepatocellular carcinoma, WTAP can promote the degradation of ETS1 through the m6A-way, which in turn promotes the progression of hepatocellular carcinoma [36]. It has also been reported that WTAP is significantly associated with tumor prognosis in gastric cancer and can influence the progression of the cancer in an immune-related manner by affecting tumor-associated T lymphocytes [37]. In addition, WTAP expression was found to be significantly higher in high-grade plasmacytoid OC than in normal cancer tissues, and it could significantly modulate tumor progression [38]. However, the molecular mechanisms through which it affects the genetic phenotype of OC are not well understood and need to be further investigated.

As is known, hypoxia is an indispensable factor in the tumor microenvironment, which arouses expression alteration of multitudinous oncogenes and tumor suppressors, thus accelerating the process of tumorigenesis [38]. In the present study, we start from the hypoxic microenvironment of OC and discover that the expression level of m6A methylase WTAP is notably upregulated in OC under hypoxia conditions (1% O_2_). In-depth studies have revealed that HIF-1*α* can mediate transcriptional activation of WTAP expression at the transcriptional level. According to previous studies in the literature, it was found that HIF contains two key factors, HIF-1*α* and HIF-2*α*, which can activate the transcriptional expression of m6A demethylase ALKBH5 gene at the transcriptional level in the development of breast cancer [39]. And it is directly by HIF-1*α* through binding with its transcription region using chip experiment in OC cell lines. WTAP mRNA and protein expression can be altered in response to changes in HIF-1*α* expression, and HIF-1*α* can positively target and regulate WTAP expression. It has been repeatedly reported that the methylation modification of m6A is a reversible process of methylation and demethylation, which is regulated by METTL3, METTL14, and WTAP, the key enzymes of m6A methylation, and FTO and ALKBH5, the enzymes of demethylation. When the dynamic balance of methylation and demethylation in the m6A methylation enzyme process is imbalanced, it can lead to pathological conditions, and in severe cases, to cancer [40]. Available literature reports that knockdown of METTL3 and METTL14 during mouse development leads to an imbalance in intracellular m6A methylation, resulting in increased levels of gene demethylation and decreased levels of methylation, thus causing loss of self-renewal ability of mouse embryonic stem cells. Recent professional research discovered the decreased tendency of m6A level in hepatocellular carcinoma after METTL14 expression inhibition [23]. Therefore, we predicted that hypoxic conditions could change the m6A methylation level in whole-cell RNA in OC with the upregulation of WTAP. In our study, we found that HIF-1*α* can significantly positively regulate the expression of WTAP in a hypoxic environment, and the high expression of WTAP in OC can promote the methylation level of m6A methylation process, so it has a very important role in the development process of OC. It can be seen that when WTAP is upregulated, the methylation process tended by m6A methylation modifications of cells is significantly increased in OC. In contrary, after shRNAs targeting WTAP, the consequences showed m6A level in RNA in OC cell lines decreased significantly. Further experiments revealed that knockdown of HIF-1*α*, which aroused WTAP upregulation, could abolish the m6A methylation effects.

Since WTAP was first discovered as a prognostic factor in glioblastoma [41], more and more research has focused on its oncogenic role in various types of malignant tumors. In cholangiocarcinoma, high WTAP expression has been reported to significantly correlate with its TNM staging, and further in vitro experiments have shown that high WTAP expression can significantly promote the migration and invasion of cholangiocarcinoma cells [42]. In addition, in pancreatic cancer, WTAP is an independent prognostic factor for the prognosis of this tumor, and it was found that the prognosis was often poor in patients with high WTAP expression [34]. In addition, in this report, a Kaplan-Meier survival analysis revealed that patients with high WTAP expression had significantly shorter survival times compared to those with low WTAP expression levels, suggesting that WTAP should be involved in the development of OC. This raised our strong concern and, at the same time, it further elucidated the important impact of WTAP in OC. We further constructed relevant stably transfected cell lines and performed the corresponding cell biological function experiments using the stably transfected cell lines. All experimental results showed that the proliferative capacity and migration and invasive ability of OC cells were significantly reduced by the decrease of WTAP expression in both in vitro and in vivo cells. Taken together, our study reported that WTAP under OC was significantly associated with the proliferation, invasion, and migration ability of cancer cells.

A large number of literature has repeatedly reported that M6A modification is not only involved in the occurrence and development of human cancer but also plays a very critical role in it. In recent studies, a new mechanism has been found that m6A modifications could regulate the maturation process of microRNA by increasing the recognition of DGCR8 with pri-miRNAs [30]. The existing literature found that the demethylase METTL3 not only significantly promotes the maturation of miRNAs such as let-7e, miR-221/222, miR-4485, miR-25, miR-93, miR-126, and miR-335 [42, 43]. Moreover, METTL14 was found to regulate the processing of miR-126 by DGCR8 mainly through a promethylation process during m6A methylation [42]. To our knowledge, this is the first comprehensive study that WTAP affected tumor formation by the regulation of the m6A modification in miRNAs.

In our study, WTAP was found to interact with microprocessor protein DGCR8 in OC by RNA immunoprecipitation experiments, and the downstream pri-miRNA was positively regulated in an m6A-dependent manner. Through the analysis of the GEO database and gene-wide expression profiling, we validated that miR-200 was significantly influenced by WTAP expression status. Subsequently, we stably disrupted WTAP expression in OC cells and in turn examined the expression of miR-200 and pri-miR-200. As expected, the expression of pri-miR-200 increased with the knockdown of WTAP, while the expression of miR-200 decreased significantly in expression. The results of miR-200 and pri-miR-200 expression examined after stable overexpression of WTAP in OC cells were opposite to the results of knockdown of WTAP expression. Moreover, when we transfected miRNA mimics into WTAP knockdown OC cells, we found that miR-200 interference could rescue the proliferation induced by WTAP in OC cells. All our findings suggest that WTAP promotes pri-miRNA maturation in OC cells through a promethylation manner during m6A methylation.

## 5. Conclusion

Our study analyzed the association between the m6A regulator WTAP and the clinical features of OC and also showed the high prognostic value of WTAP for OC. We also found that the hypoxic microenvironment drives the biological process of OC malignant progression by affecting the glycolytic pathway of cancer cells, in which WTAP regulates the miR-200 that can regulate the key gene HK2 in the the pathway by interacting with DGCR8, a key component of the canonical microprocessor complex of microRNA biogenesis. In short, this study provides a new marker for assessing the prognosis of OC.

## Figures and Tables

**Table 1:** Correlation of WTAP expression with patient's clinical and pathological characteristics.

Item		Low expression	High expression	Total	*P* value
Age	>55	50	60	110	0.9
	≤55	71	95	166	
	Total	121	155	276	
Histologic subgroups	Serous	55	88	143	0.013
	Clear cell	11	13	24	
	Endometrioid	16	26	42	
	Mucinous	39	28	67	
	Total	121	155	276	
FIGO stage	I	78	92	170	0.001
	II	24	17	41	
	III-IV	19	46	65	
	Total	121	155	276	
Lymph node status	—	115	133	248	0.02
	+	6	22	28	
	Total	121	155	276	
Grade of serous	Low	23	33	56	0.001
	High	32	55	87	
	Total	55	88	143	

## Data Availability

The data used to support the findings of this study are included in the article. Further inquiries can be directed to the corresponding author.

## Supplementary Materials

Supplementary Materials Supplementary Figure 1: silencing of WTAP suppresses ovarian cancer cell proliferation capacity in vitro and tumorigenesis in vivo. (A) WTAP knockdown efficiency was confirmed by RT-PCR in A2780 and 3AO cells. (B) The cell proliferation of the Nc and sh-groups in A2780 and 3AO cells was evaluated by CCK8 assay at 0, 24, 48, 72, and 96 h. The results revealed that knockdown of WTAP significantly inhibited the proliferation of A2780 and 3AO cells in vitro (*P* < 0.01). (C) Plate colony formation assay of A2780/WTAP-sh and 3AO/WTAP-sh and Nc cells on regular culture plates after 14 days of culture. Relative colony numbers of WTAP-sh cells were significantly decreased than that of Nc cells. (D) Photographs of tumors from mice inoculated with A2780-Nc and A2780-sh2 cells. (E and F) Tumor growth curve and tumor weight of Nc and sh2 groups are shown in figure4E-4F, *n* = 5. Supplementary Figure 2: overexpression of WTAP promotes ovarian cancer cell proliferation, migration, and invasion in vitro. (A) Overexpressed WTAP in H8901, OVCAR8 cells and the overexpression efficiency were confirmed by RT-PCR. (B) CCK8 experiments in WTAP-overexpressing group compared with the oevec group in both H8901 and OVCAR8 cells. (C-F) Transwell migration and invasion assays in the WTAP-overexpressing group compared with the oevec group in both H8901 and OVCAR8 cells. Original magnifification: 200x. Quantifications of cells on the lower surface of the membrane were performed with three randomly chosen fields and demonstrated on the right panels. (G and H) Wound healing assay in WTAP-overexpressing cells. The scratch was measured 24 hours later. The cell boundary was outlined by the dotted black line. Quantification of wound healing percents was analyzed in H8901 and OVCAR8 cells, respectively. Data are means ± SD (^∗^*P* < 0.05, ^∗∗^*P* < 0.01). Supplementary Figure 3: MiR200 overexpression rescues the proliferation and migration capacity induced by WTAP in OC cells. (A) MiR200 is highly expressed in tumor tissues than in normal tissues in OC. (B) MiR200 mimics could partly increase the proliferation capacity inhibited by the knockdown of WTAP in both A2780 and 3AO cells. (C and D) MiR200 mimics could partly increase the cell migration capacity induced by WTAP inhibition in both A2780 and 3AO cells. Supplementary Figure 4: schematic representation of the nuclear role of WTAP in miRNA processing.
