# Supplementary material for: HIF-1α Regulated WTAP Overexpression Promoting the Warburg Effect of Ovarian Cancer by m6A-Dependent Manner
Source: J Immunol Res. 2022 Jun 12;2022:6130806. doi: 10.1155/2022/6130806 (PMC9207020; doi:10.1155/2022/6130806)
Supplement: Supplementary Materials — Supplementary Figure 1: silencing of WTAP suppresses ovarian cancer cell proliferation capacity in vitro and tumorigenesis in vivo. (A) WTAP knockdown efficiency was confirmed by RT-PCR in A2780 and 3AO cells. (B) The cell proliferation of the Nc and sh-groups in A2780 and 3AO cells was evaluated by CCK8 assay at 0, 24, 48, 72, and 96 h. The results revealed that knockdown of WTAP significantly inhibited the proliferation of A2780 and 3AO cells in vitro (P < 0.01). (C) Plate colony formation assay of A2780/WTAP-sh and 3AO/WTAP-sh and Nc cells on regular culture plates after 14 days of culture. Relative colony numbers of WTAP-sh cells were significantly decreased than that of Nc cells. (D) Photographs of tumors from mice inoculated with A2780-Nc and A2780-sh2 cells. (E and F) Tumor growth curve and tumor weight of Nc and sh2 groups are shown in figure4E-4F, n = 5. Supplementary Figure 2: overexpression of WTAP promotes ovarian cancer cell proliferation, migration, and invasion in vitro. (A) Overexpressed WTAP in H8901, OVCAR8 cells and the overexpression efficiency were confirmed by RT-PCR. (B) CCK8 experiments in WTAP-overexpressing group compared with the oevec group in both H8901 and OVCAR8 cells. (C-F) Transwell migration and invasion assays in the WTAP-overexpressing group compared with the oevec group in both H8901 and OVCAR8 cells. Original magnifification: 200x. Quantifications of cells on the lower surface of the membrane were performed with three randomly chosen fields and demonstrated on the right panels. (G and H) Wound healing assay in WTAP-overexpressing cells. The scratch was measured 24 hours later. The cell boundary was outlined by the dotted black line. Quantification of wound healing percents was analyzed in H8901 and OVCAR8 cells, respectively. Data are means ± SD (∗P < 0.05, ∗∗P < 0.01). Supplementary Figure 3: MiR200 overexpression rescues the proliferation and migration capacity induced by WTAP in OC cells. (A) MiR200 is highly expressed in tum [file 6130806.f1.pdf]

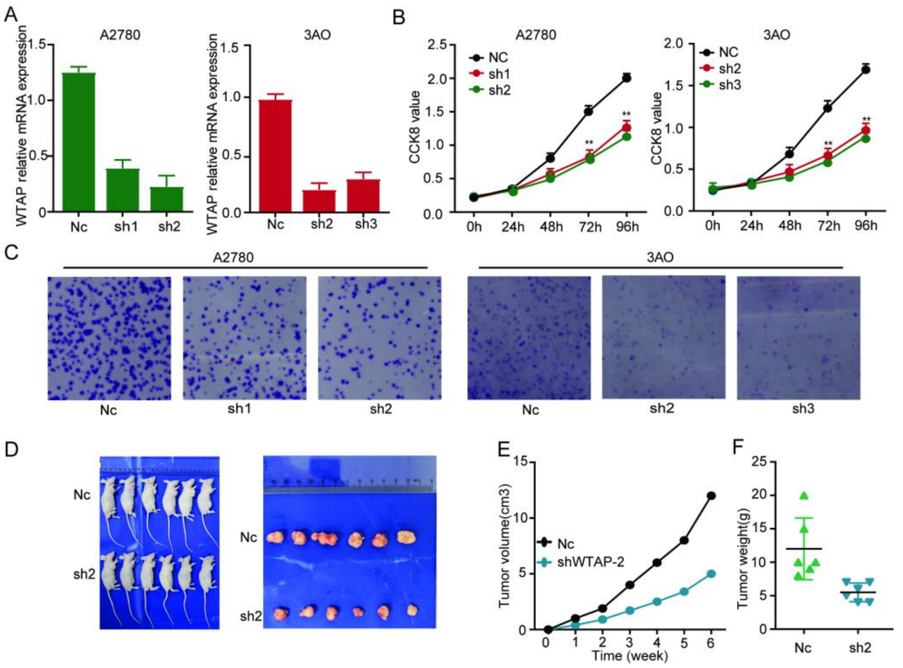

Supplementary figure 1 Silencing of WTAP suppresses ovarian cancer cell proliferation capacity in vitro and tumorigenesis in vivo

(A) WTAP knockdown efficiency was confirmed by RT-PCR in A2780 and 3AO cells. (B) The cell proliferation of Nc and sh-groups in A2780 and 3AO cells were evaluated by CCK8 assay at 0, 24, 48, 72, 96h. The results revealed that knockdown of WTAP significantly inhibited the proliferation of A2780 and 3AO cells in vitro ($P<0.01$). (C) Plate colony formation assay of A2780/WTAP-sh and 3AO/WTAP-sh and Nc cells on regular culture plates after 14 days of culture. Relative colony numbers of WTAP-sh cells were significantly decreased than that of Nc cells. (D) Photographs of tumors from mice inoculated with A2780-Nc and A2780-sh2 cells. (E-F) Tumor growth curve and tumor weight of Nc and sh2 groups were shown in figure4E-F, n = 5.

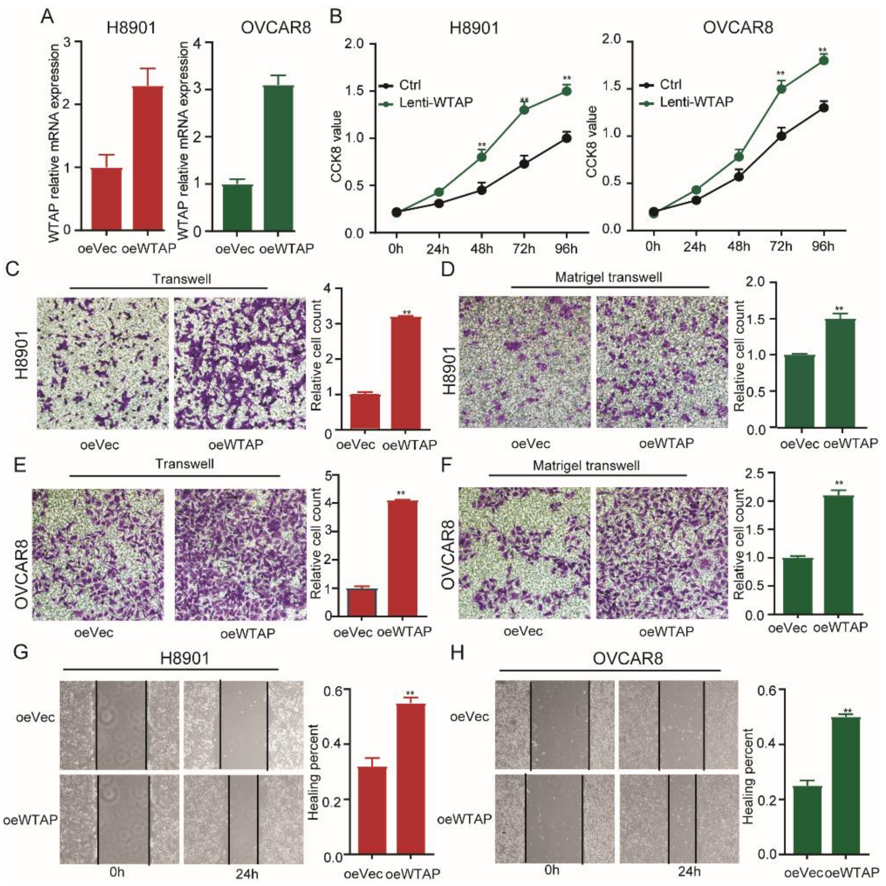

Supplementary figure 2 Overexpression of WTAP promotes ovarian cancer cell proliferation, migration and invasion in vitro.

(A) Overexpressed WTAP in H8901, OVCAR8 cells and the overexpression efficiency was confirmed by RT-PCR. (B) CCK8 experiments in WTAP-overexpressing group compared with oevec group in both H8901 and OVCAR8 cells. (C-F) Transwell migration and invasion assays in WTAP-overexpressing group compared with oevec group in both H8901 and OVCAR8 cells. Original magnification: 200×. Quantifications of cells on the lower surface of the membrane were performed with three randomly chosen fields and demonstrated on the right panels. (G-H) Wound healing assay in WTAP-overexpressing cells. The scratch was measured 24 hours later. The cell boundary was outlined by the dotted black line. Quantifification of wound healing percents was analyzed in H8901, OVCAR8 cells respectively. Data are means ± SD (*$P < 0.05$, **$P < 0.01$).

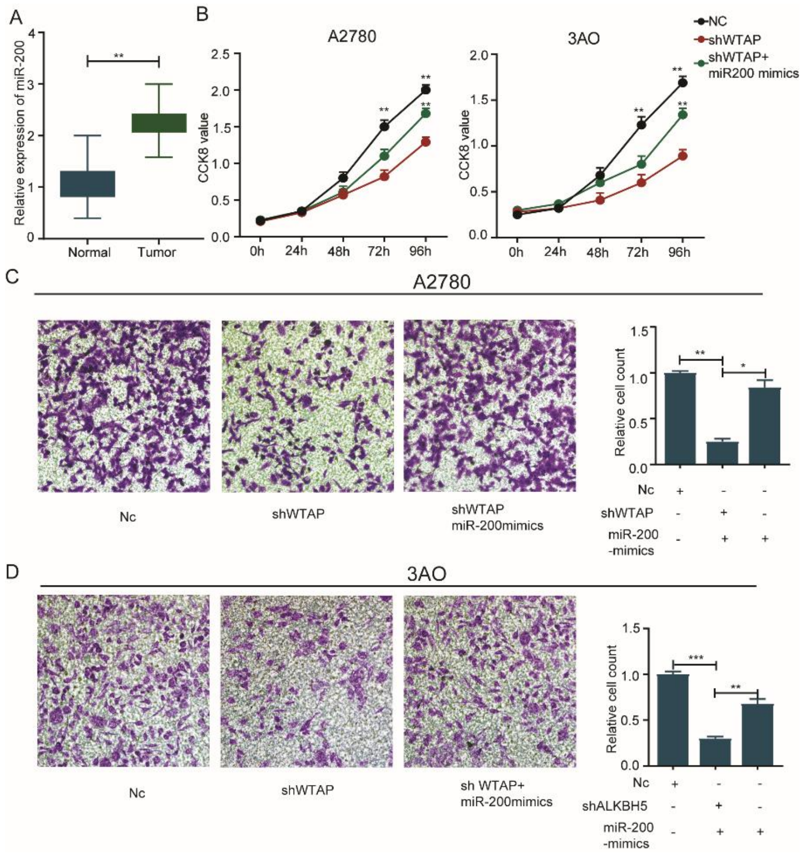

Supplementary figure 3

MiR200 overexpression rescues the proliferation and migration capacity induced by WTAP in OC cells

(A) MiR200 is highly expressed in tumor tissues than in normal tissues in OC. (B) MiR200 mimics could partly increase the proliferation capacity inhibited by the knockdown of WTAP in both A2780 and 3AO cells. (C-D) MiR200 mimics could partly increase the cells migration capacity induced by WTAP inhibition in both A2780 and 3AO cells

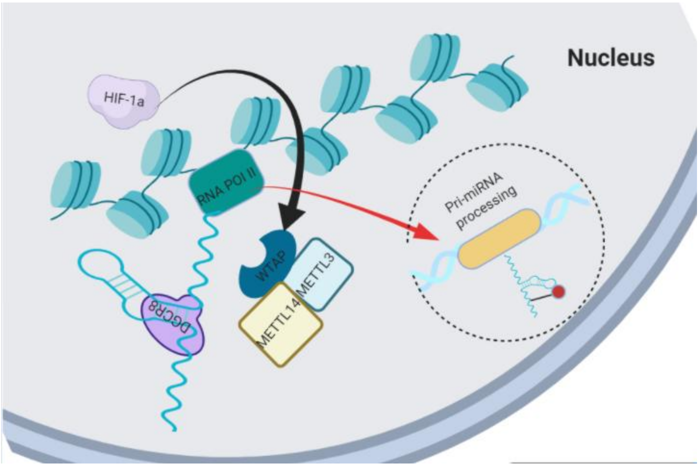

Supplementary figure 4

Schematic representation of the nuclear role of WTAP in miRNA processing .
